# The narratives of war (NoW) corpus of written testimonies of the Russia-Ukraine war

**DOI:** 10.1007/s10579-025-09813-8

**Published:** 2025-02-19

**Authors:** Serhii Zasiekin, Larysa Zasiekina, Emilie Altman, Mariia Hryntus, Victor Kuperman

**Affiliations:** 1 University College London, London, UK; 2 Lesya Ukrainka Volyn National University, Lutsk, Ukraine; 3 University of Exeter, Exeter, UK; 4 McMaster University, Hamilton, Canada

**Keywords:** The Narratives of War (NoW) corpus, The Russia-Ukraine war, Civilians, War-related experience, Psychological impact, Open-vocabulary linguistic analysis

## Abstract

Documentation and analysis of psychological states experienced by witnesses and survivors of catastrophic events is a critical concern of psychological research. This paper introduces the new corpus of written testimonies collected from nearly 1500 Ukrainian civilians from May 2022–January 2024, during Russia’s invasion of Ukraine. The texts are available in the original Ukrainian and the English translation. The Narratives of War (NoW) corpus additionally contains demographic and geographic data on respondents, as well as their scores in tests of PTSD symptoms and moral injury. The paper provides a detailed introduction into the method of data collection and corpus structure. It also reports a quantitative frequency-based “keyness” analysis that identifies words particularly representative of the NoW corpus, as compared to the reference corpus of Ukrainian texts that predates the war with Russia. These key words shed light on the psychological state of witnesses of war. With its materials collected during the ongoing war, the corpus contributes to the body of knowledge for studies of the psychological impact of war and trauma on civilian populations.

Documentation and analysis of psychological states experienced by witnesses and survivors of catastrophic events is a critical concern of psychological research. At the time of this writing, Russia’s invasion of Ukraine has lasted over 1000 days. This ongoing war has displaced over 6 million Ukrainians (UNHCR, 2023) and exposed many more to extreme violence, abuse, deprivation, and loss. These events are projected to have a devastating impact on the mental health of Ukrainians and give rise to collective intergenerational trauma at a genocide scale (Alibudbud, 2022; Baum, 2008; Bürgin et al., 2022; Cai et al., 2022; Chaaya et al., 2022; Junior et al., 2022; Konstantinov et al., 2022; Patel & Erickson, 2022). While many methods exist to assess mental well-being and trauma, this paper focuses on the psycholinguistic analysis of language productions by Ukrainian civilians. This paper introduces a new corpus of written productions and psychological data collected from Ukrainians from May 2022–January 2024 and presents examples of linguistic analyses that such data make possible.

This paper pursues two related goals. One is to introduce to the research community a new corpus of texts written by Ukrainians during Russia’s ongoing invasion of Ukraine. The corpus–labeled *Narratives of War* (or NoW)–presents short written narratives along with demographic and psychological data collected online from over 2000 Ukrainians. This paper describes the annotations and supplementary information included in the corpus with the purpose of making it usable for a broad variety of research purposes and questions related to the experience of war in a non-combatant population. Along with basic demographic information (gender, age, marital status, highest education level attained, experience of collective trauma in family history and others), the corpus reports whether the participant was forcibly displaced (within Ukraine or abroad) and whether their pre-war region of residence was occupied at the time of task completion. Finally, the corpus incorporates responses to two psychological instruments that tap into symptoms of post-traumatic stress disorder (PTSD) and moral injury. One of the defining features of the NoW corpus is that it is being collected during wartime and thus represents immediate first-hand accounts of war-related experiences. This contrasts with the much more common situation where written accounts of war are produced in retrospection—years if not decades after the events they describe, becoming subject to self-reflection, forgetting, and self-editorializing (see review in Altman & Kuperman, 2023). This lived experience expressed in language provides unique insights and perspectives on the psychological effects of war on civilians. With the broad geographic coverage from all major regions of Ukraine, and a temporal coverage beginning some 7 weeks after Russia’s invasion (17 May, 2022) and up to almost two years since the invasion (14 January, 2024), the corpus is a valuable resource for research on the psychological impact of war.

The second goal of the paper is to gain insight into the collective psychological state of the Ukrainian witnesses of Russia’s invasion. The notion that patterns of language use are indicative of the psychological state of the speaker harks back to at least as early as Freudian psychoanalysis (e.g., Freud, 1989/1901). In the modern-day science, the systematic analysis of language use has uncovered words and word combinations that are diagnostic and predictive of such diverse psychological and mental states as suicidal ideation, dementia, depression, and other affective disorders, to name a few (see reviews by Boyd & Schwartz, 2021 and Pennebaker et al., 2003). Additionally, linguistically expressed integrative complexity reveals resources for survival and successful postwar adaptation (Suedfeld et al., 1998).

Individual and group differences in non-pathological states and traits appear to have a distinct expression in the word choice and morpho-syntactic features of language use as well, including personality type, age, gender, political ideology, and belief system (e.g., Schwartz et al., 2013; Tausczik, & Pennebaker, 2010). In sum, this prior literature suggests that an analysis of language used by witnesses of the ongoing war can be indicative of their defensive, emotional and psychological responses to the events. While we harvest the NoW corpus data to pursue only a narrow range of questions, we state other uses of these data throughout the paper.

Existing research highlights several methods of detecting meaningful patterns of language use in a corpus of texts and correlating those patterns with psychological variables (see recent review by Eichstaedt et al., 2021). The approach we adopt is the open-vocabulary linguistic analysis of natural texts, which operates on all words in all texts of a corpus. This contrasts with an alternative closed-vocabulary method that relies on finite and typically small, pre-determined and curated word lists (e.g., Pennebaker et al., 2003; Stirman & Pennebaker, 2001; Tausczik & Pennebaker, 2010): see Zasiekin et al. (2022), Zasiekina et al. (2019) for application of the closed-vocabulary LIWC instrument to Facebook entries written by Ukrainian civilians in 2022–23. The specific open-vocabulary approach that we take is the keyness analysis (Monroe et al., 2008). It involves (i) calculating the frequency of occurrence of every n-gram (words or combinations of *n* words) in the target corpus and (ii) comparing the resulting frequency values with the corresponding values for the n-grams occurring in a reference corpus that serves as a control. In the present paper, the target corpus is NoW and the reference corpus is of a similar genre to the target (consisting of blog entries and interviews in Ukrainian) but collected prior to 2014 (see justification below). The frequency comparison—explained in detail below—identifies n-grams that are most predictive of the narratives recorded during Russia’s invasion of Ukraine, compared to previous historical periods. Knowing the lexical items that are key to the corpus offers an insight into the objects, concepts, topics, and beliefs that accompany the psychological state of the war witnesses (e.g., Altman & Kuperman, 2023).

The structure of the paper is as follows. We begin with spelling out the method of creating the NoW corpus and its contents, and we follow up with the keyness analysis of the NoW texts as compared to the reference pre-war corpus of a similar genre.

## Methods

### Participants

This first release of the Narratives of War (NoW) includes data collected between May 17, 2022, and January, 14, 2024, i.e., 82 and 691 days since the beginning of Russia’s invasion of Ukraine on February 24, 2022. In that time period, 2208 participants completed the narrative writing task and the demographic questionnaire. Additionally, 2060 of those participants completed psychological questionnaires, see descriptions below. Each participant submitted data only once. The project was reviewed and approved by the Research Ethics Committee (#03-24/04/1070) at Lesya Ukrainka Volyn National University, Ukraine and the Research Ethics Board (#6045) of McMaster University, Canada.

The respondents ranged in age from 12 to 86 years and represented all administrative geographic units (Oblasts) of Ukraine. We did not ask whether the participant was in active military service for safety and confidentiality reasons, yet the vast majority of the sample are likely civilians. We removed from further consideration participants younger than 18 years old, participants who did not consent to the use or publication of their data, as well as participants with duplicate or plagiarized narratives or with invalid age values. Analyses below are based on the resulting pool of 2003 participants with valid narratives, demographic data and psychological questionnaire responses. Table 1 summarizes the descriptive statistics of the demographic and questionnaire data, as well as the NoW corpus.

**Table 1:** Descriptive statistics of the NoW corpus of texts and the participant sample

Variable	Values
N participants	2003
N word tokens (minus stopwords)	666,912 (330,493)
N distinct word types (minus stopwords)	37,067 (36,725)
N distinct lemmas (minus stopwords)	20,322 (20,087)
Mean narrative length, words	Range = 1:2324, M = 333, SD = 237
Gender	Men: 220 (16%) Women: 1152 (84%) Other: 0
Age	Median = 32; range = 18:86
Highest completed education	Secondary: 439 College: 564 Bachelor: 314 Master or PhD: 686
Marital Status	Single: 1022 Married: 961 In a relationship: 20
Forcibly displaced	Yes: 858 (43%) No: 1146
Occupation of region of pre-war residence at the time of writing	Never occupied: 703 Occupied: 976 De-occupied: 324
Presence of intergenerational family trauma	Holocaust: 22 Holodomor: 301 None: 1220 Other: 470
PCL-5 test scores (PTSD symptoms)*	Range = 0:80, M = 41, SD = 19
MISS test scores (Moral Injury)*	Range = 10:92, M = 42, SD = 17

*Based on 1225 participants who completed the PCL-5 and MISS questionnaires

### Procedure

The study was administered online. Participants were recruited by the Ukrainian Psychotrauma Center at Lesya Ukrainka Volyn National University (Lutsk, Ukraine) via its website and Facebook page and were provided with an information sheet and a consent form. All instructions were given in Ukrainian. Participants gave consent for their data to be analyzed and published in de-identified form by filling the respective field in the online Google form. Upon consent, participants were invited to fill in their demographic data and questionnaires through Google forms. They were also asked to complete the narrative writing task. The open-ended instruction was to “describe their experience of the Russia-Ukraine war in at least 300 words”. Participants were paid UAH200 as a compensation for their time.

### Materials and variables

Written narratives on the war experience were typed into the text box in the Google form. There were no space or time limits imposed on the writers for completing this or other tasks. Twelve participants wrote their narratives in Russian, and the rest wrote in Ukrainian: For uniformity, Russian narratives were translated into Ukrainian by native speakers of Ukrainian, professional translators with experience, with all the essentials preserved, i.e. both their content and style, along with pragmatics, and analyzed in this form. The translation adequacy was confirmed by a group of experts in translation.

The demographic questionnaire consisted of free-form short answers or drop-down menus with multiple choice questions. It included questions about the participant’s age, gender, marital status, and highest completed education, as well as displacement status (not displaced, displaced internally, or displaced abroad) and administrative unit (Oblast) of their pre-war residence. The latter variable was further coded into “never occupied”, “occupied at the time of writing”, “de-occupied at the time of writing” categorical levels. Additionally, participants provided a free-form response to the question of whether there was a collective trauma (e.g., the Holodomor or Holocaust) in their family history. Table 1 summarizes the distribution of the variables in the sample.

The study made use of two psychological questionnaires, both administered in Ukrainian. The standard PTSD Checklist for DSM-5 (Weathers et al., 2013), labeled here as PCL-5, consists of 20 Likert-scale questions asking how much the respondent has been bothered by a given problem in the past month, from 0 (“not at all”) to 4 (“extremely”). The PCL-5 total scores range from 0 to 80, with a higher score corresponding to a higher total severity of PTSD symptoms. The second questionnaire is the Military Version Short Form of the Moral Injury Symptom Scale (Koenig et al., 2018), labeled here as MISS. This Likert scale consists of 10 statements (e.g., “I feel betrayed by leaders who I once trusted”) with response options ranging from 0 (“strongly disagree”) to 10 (“strongly agree”). The total score occupies a range from 10 to 100, with higher values corresponding to a higher level of moral injury. Zasiekina et al.’s (2023) analyses of the test scores in the NoW corpus indicated a very high reliability of the PCL-5 scores (ICC(2,*k*) = 0.92, 95% CI [0.91, 0.92]), in agreement with Shevlin et al.’s (2018) earlier pre-war work on internally displaced Ukrainians. However, reliability of the MISS scores was only moderate (ICC(2,*k*) = 0.53, 95% CI [0.47, 0.59]).

All analyses in this paper were conducted using the statistical platform R v 4.2.2 (R Core Team, 2022). Plots were made using the package *ggplot2* (Wickham, 2011).

## Results

The corpus of written narratives from 1372 participants underwent minor proof-reading to correct for spelling errors and was translated by professional translators from Ukrainian to English, to ensure accessibility for a broad research community. Both the Ukrainian and the English versions were further de-identified, e.g., personal names and nicknames were replaced by the tag [NAME] and names of geographic locations (except for names of regions and major cities) by the tag [CITY]. If multiple personal or geographic names were used, they were replaced by the tags [CITY1], [CITY2] throughout the narrative. The outcome of this step is a set of de-identified written narratives supplemented by demographic and psychological data from their authors.

The next step was to apply natural language processing to the narratives in Ukrainian. The narratives were tokenized (i.e., separated into individual words and punctuation), tagged for part-of-speech (e.g., noun, verb, preposition, etc.) and morphological information (e.g., number, grammatical gender and case and animacy for nouns). They were also lemmatized (e.g., relating each word to its dictionary form or lemma, e.g., relating *work*, *worked*, *working* and *works* to lemma *work*) and syntactically parsed (syntactic dependencies were identified between words and phrases). This processing was achieved by using the UDpipe version 2.0 implemented in the R package udpipe, version 0.8.11 (Straka & Straková, 2017) with a pretrained Ukrainian model, version 2.10–220711 (additional information is provided at https://ufal.mff.cuni.cz/udpipe). The outcome of this step is a word-by-word representation of the corpus of narratives, supplemented by extensive morpho-syntactic information.

This paper releases the materials of the NoW corpus in two formats. One format is organized around individual narratives as a unit of analysis. This format includes the demographic and psychological data from participants, as well as their narratives, in the Ukrainian language and in the English translation. Reported variables include: the number of the narrative (e.g., doc1 for narrative 1); age, gender, and marital status of the participant; region of residence before the full-scale war in 2022; occupation status of the region at the time of writing (occupied, not occupied, previously occupied but liberated); displacement status (yes/no); highest level of education; presence of the collective trauma in family history; date of submission; the narrative; and scores in the PCL-5 and MISS questionnaires. Another format of presentation has individual words from each narrative as a unit of analysis. It only reports Ukrainian data and contains the following variables: number of the narrative, paragraph number, sentence number, sentence, word number, word, lemma, part-of-speech tag, morphological parsing, and syntactic role of the word in the sentence. The two formats are linked by the first column in respective files, which contains the narrative’s ordinal number in the corpus. The narrative-based format has a sample size of 2003 participants, and the word-based format contains data on 666,912 word tokens, see Table 1 for details and the Data Availability section for access.

### Keyness analysis

A further trimming step was applied to the NoW corpus to enable a meaningful comparison to the reference corpus. We removed all punctuation and function words (pronouns, prepositions, and other closed-class words) from the Ukrainian texts, using a custom-made list of stopwords in Ukrainian, see descriptive statistics following this removal in Table 1. As the next step, we calculated the frequency of occurrence of each word in the entire NoW corpus: Lemmas (or dictionary word forms) were used for this purpose to account for morphological variants of the same word.

The reference corpus, against which frequency estimates from the NoW corpus are compared, is the EGO component of the General Regionally Annotated Corpus of Ukrainian, version 17 (GRAC, Shvedova, 2020). The EGO component “features memoires, letters and diaries, including a considerable corpus of Facebook posts representing blogs of people from all the Ukrainian regions and from the diaspora” (http://uacorpus.org/Kyiv/en/rozmitka-tekstiv/stili-tematika-i-zhanri). We selected this genre space of the GRAC corpus because it comes closest to the personal narratives elicited in the course of our study. We restricted the origin of the text to Ukraine only (excluding the diaspora), and to interviews and memoires. Furthermore, we restricted the date range to 1991–2013, a period that begins with establishment of Ukraine as an independent state and ends before the Russian annexation of Crimea in 2014 and the subsequent invasion of Donetsk and Luhansk Oblasts. The selection of the time period outside of wartime does not of course imply that the respective documents are free of the mention of previous wars. In fact, the analyses below suggest that World War II is present in the discourse of Ukrainians prior to 2014. This makes the comparison of this reference corpus to the NoW corpus all the more intriguing, because it can point to the concepts and themes that come to the forefront of the lived experience of the war versus experiences of peacetime, which include generational memories of the war.

The GRAC interface makes available the frequency list of lemmas found in the EGO component of this reference corpus in the given time period. It does not provide either the original texts, nor the frequency lists of bigrams or trigrams (duplets or triplets of adjacent words). For this reason, our keyness analysis is restricted to single words (unigrams). The word frequency list from the GRAC corpus contained 50,139 unique lemmas.

The next step combined the word frequency lists from the NoW and GRAC corpora. If a word from one corpus was not found in the other frequency list, it was assigned a frequency of zero. The combined list contained 59,190 lemmas. The keyness analysis based on this comparative list highlights which words are particularly diagnostic and representative of the target corpus versus the reference corpus. Specifically, the analysis produces a numeric keyness score, which quantifies how unique each word is to the target corpus (NoW), relative to the reference corpus (GRAC EGO). To calculate keyness, we used the log-odds ratio informative Dirichlet prior (LORIDP) as proposed by Monroe et al. (2008) and validated in Schmidtke and Kuperman (2017) and Altman and Kuperman (2023).

LORIDP estimates the difference in relative frequency for each lemma *w* between the NoW corpus (*i*) and the reference EGO corpus (*j*). LORIDP operationalizes this difference as the log-odds ratio, ${\delta }_{w}^{\left(i-j\right)}$, which is calculated as:

$${\delta }_{w}^{\left(i-j\right)}= log\left(\frac{{y}_{w}^{i}+{\alpha }_{w}}{{n}^{i}+{\alpha }_{0}-\left({y}_{w}^{i}+{\alpha }_{w}\right)}\right)-log\left(\frac{{y}_{w}^{j}+{\alpha }_{w}}{{n}^{j} + {\alpha }_{0} - \left({y}_{w}^{j}+{\alpha }_{w}\right)}\right)$$

In this case, ${y}_{w}^{i}$ is the frequency count of lemma *w* in the NoW corpus, and ${y}_{w}^{j}$ is the frequency count of lemma *w* in the EGO corpus; ${n}^{i}$ is the total number of lemma tokens in the NoW corpus, and ${n}^{j}$ is the total number of lemma tokens in the EGO corpus. ${\alpha }_{0}$ is the sum of all lemma tokens in the NoW and EGO corpora jointly, and ${\alpha }_{w}$ is the summed count of lemma *w* in both corpora.

Next, the variance for the log-odds ratio is estimated using the equation:

$$ \sigma^{2} \left( {\delta_{w}^{{\left( {i - j} \right)}} } \right) \approx \frac{1}{{y_{w}^{i} + \alpha_{w} }} + \frac{1}{{y_{w}^{j} + \alpha_{w} }} $$

With the log-ratio and its variance, we calculated the standardized z-value of the LORIDP for each lemma:

$$z=\frac{{\updelta }_{w}^{\left(i-j\right)}}{\sqrt{{\upsigma }^{2}\left({\updelta }_{w}^{\left(i-j\right)}\right)}}$$

**Fig. 1:**
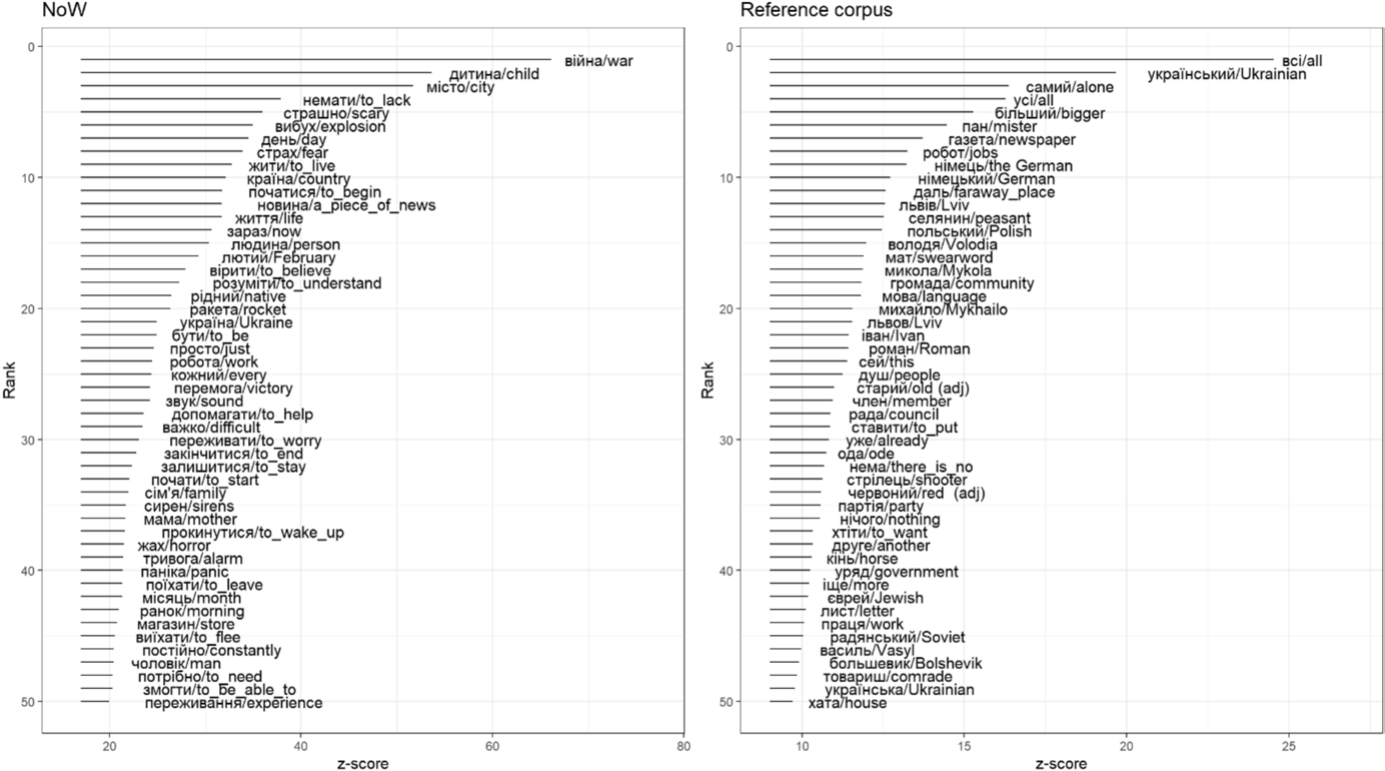
Words with the highest keyness z-scores: Fifty most diagnostic words for the NoW corpus (left panel) and for the EGO corpus (right). Absolute values of keyness z-scores are shown on the x-axes and ranks on the y-axes. Words are given in the Ukrainian original and the English translations

The resulting standardized z-value stands for the keyness score of each word. Greater positive z-values signal words that are more diagnostic of the narratives of the Russia-Ukraine war, while greater negative values are underrepresented in that target corpus and are more representative of the language used in the reference corpus, in the period preceding this war. Figure 1 below illustrates words most and least diagnostic of the Narratives of War corpus, as compared to the pre-war reference corpus. The words are presented separately for each corpus and ranked by the absolute values of their z-scores. English translations are provided along with Ukrainian originals: In a few cases of polysemy or homonymy (e.g., тpивoгa can be translated either as anxiety or alarm) we chose the translation that was more appropriate given the concordance contexts in which the word appeared in the NoW corpus (e.g., alarm).

Since comparisons in relative frequency are applied to a list of thousands of lemmas, it is necessary to control for multiple comparisons and inflation of the Type I error. To this end, we applied the Bonferroni family-wide correction to keyness z-scores. Our LORIDP threshold for statistical significance of the z-score for a given lemma was calculated as |*z*|= 5.10, corresponding to *p* < 0.01/59190. After removing lemmas that did not meet the statistical significance threshold, we were left with 726 lemmas that are diagnostic of lexical use in the NoW corpus, and 455 lemmas that are diagnostic of the EGO corpus: The list is provided in the Supplementary materials.

Figure 1 presents the top 50 Ukrainian words diagnostic of the NoW corpus (left) and 50 words diagnostic of the reference EGO corpus (right). The words are ranked in the order of their keyness (with rank 1 marking the word most diagnostic of the respective corpus).

Unsurprisingly, NoW texts overrepresented words related to realities of the ongoing armed conflict: war, explosion, rocket, bomb shelter, alarm, and 24 February (the first day of the full-scale invasion in 2022). A large number of words diagnostic of the wartime corpus conveyed emotional states: e.g., anxiety, fear, scary, horror, stress, hard, and panic. Most of the emotional states are highly negative, but exceptions occur as well (victory, believe, life). Family terms were also commonly found: child, family, children, daughter, and house. They point to topics and tropes that are at the forefront of the minds of Ukrainian witnesses of the war.

Words that are under-represented in the NoW corpus compared to the pre-war reference EGO corpus contain several names of geographic locations (Lviv) and personal names (Mykhailo, Mykola, Volodya). The reason is that such names were cleaned out of the NoW corpus for confidentiality reasons and replaced with labels like CITY1 or NAME1. Many other words were generic and not skewed towards any specific topic or emotional state.

In sum, the open-vocabulary method of keyness analysis points to biases in the word choices that paint a consistent and psychologically meaningful picture of the mindset of participants. Their attention is drawn to the realities of the ongoing war with primary focus on family, specifically on children as one of the most vulnerable groups during the war (Júnior et al., 2022; Matiashova et al., 2022). Emotional states are mainly represented by anxiety, stress, panic, and fear: These emotions are the most common responses to traumatic stress with fear and terror as the basic emotional markers of PTSD (Amstadter et al., 2008). Another observation of interest is the absence of time-related terms among the key words for the NoW corpus, whereas the reference EGO corpus contains temporal terms like “year” and “summer”. The decrease in frequency for words expressing time is a robust marker of PTSD symptoms (Rahman & Brown, 2021).

As the corpus grows over time, future analyses will identify the temporal dynamics of positive and negative emotions, which may go in lockstep with the course of the war. Future research will also directly link linguistic analyses of NoW texts to the psychological data on PTSD symptoms and moral injury collected from participants. Additional dimensions of major interest include the influence of displacement and occupation on the psychological state of participants and the impact of collective trauma in the participant’s family history, as revealed through their written texts and questionnaire responses.

## Data Availability

The data of the NoW corpus and the code and data used in the analyses reported above are available at https://osf.io/pz958/
